# Lag synchronization of coupled time-delayed FitzHugh–Nagumo neural networks via feedback control

**DOI:** 10.1038/s41598-021-82886-x

**Published:** 2021-02-16

**Authors:** Malik Muhammad Ibrahim, Muhammad Ahmad Kamran, Malik Muhammad Naeem Mannan, Il Hyo Jung, Sangil Kim

**Affiliations:** 1 Department of Mathematics, Pusan National University, Busan, 46241 Republic of Korea; 2 Department of Cogno-Mechatronics Engineering, Pusan National University, Busan, 46241 Republic of Korea; 3 School of Allied Health Sciences, Griffith University, Gold Coast, QLD 4222 Australia

**Keywords:** Neuroscience, Neurology, Mathematics and computing

## Abstract

Synchronization plays a significant role in information transfer and decision-making by neurons and brain neural networks. The development of control strategies for synchronizing a network of chaotic neurons with time delays, different direction-dependent coupling (unidirectional and bidirectional), and noise, particularly under external disturbances, is an essential and very challenging task. Researchers have extensively studied the synchronization mechanism of two coupled time-delayed neurons with bidirectional coupling and without incorporating the effect of noise, but not for time-delayed neural networks. To overcome these limitations, this study investigates the synchronization problem in a network of coupled FitzHugh–Nagumo (FHN) neurons by incorporating time delays, different direction-dependent coupling (unidirectional and bidirectional), noise, and ionic and external disturbances in the mathematical models. More specifically, this study investigates the synchronization of time-delayed unidirectional and bidirectional ring-structured FHN neuronal systems with and without external noise. Different gap junctions and delay parameters are used to incorporate time-delay dynamics in both neuronal networks. We also investigate the influence of the time delays between connected neurons on synchronization conditions. Further, to ensure the synchronization of the time-delayed FHN neuronal networks, different adaptive control laws are proposed for both unidirectional and bidirectional neuronal networks. In addition, necessary and sufficient conditions to achieve synchronization are provided by employing the Lyapunov stability theory. The results of numerical simulations conducted for different-sized multiple networks of time-delayed FHN neurons verify the effectiveness of the proposed adaptive control schemes.

## Introduction

Synchronization plays a tremendous role in information transfer and decision-making in different fields of science and technology^1^. In the past decade, many researchers have investigated different synchronization schemes, methodologies, and regimes for chaotic systems such as lag synchronization^2^, generalized synchronization^3^, phase synchronization^4^, projective synchronization^5^, anticipating synchronization^6^, cluster synchronization^7^, and consecutive synchronization^8^. Lag synchronization, introduced for drive and response systems and extensively studied, is defined as an overlapping of the shifted-in-time states of two systems with a positive constant.

In neuroscience, the interactions of neurons and their networks have been the primary targets for investigating the differences in the functionality and dynamics of healthy and diseased brains^9–11^. For example, the brain responses of stroke patients have been analyzed and compared with those of healthy subjects to develop an effective rehabilitation system^12,13^. Neurons in the brain are connected to other neurons in the same or other regions to form a network of neurons for communication and efficient information processing. In the past decades, the internal dynamics behind the coordination and communication mechanism of neurons and their networks have been explored by developing different strategies and systems^14–18^. The processing of cognitive information in the brain is based upon the synchronized interactions between large numbers of neurons distributed within and across different specialized brain regions^19^. Researchers have found that synchronization between individual neurons or networks of neurons is an important factor for the stable and efficient working of the brain^20,21^. Experimental and theoretical results from previous studies suggested that synchronization of neuronal activity is not only a major property of cortical and subcortical networks within and across different brain regions but also helps to perform many functions in cognitive processes^19^. Therefore, synchronization can be considered as the basis for signal transmission and processing in both healthy and abnormal brains^20^. For instance, previous studies found synchronization in the brain regions including hippocampal and olfactory^22^, and it has been reported that brain disorders and disorder in body functionalities such as heart rhythm and gait could be caused by the absence of synchronization between neurons^23^. Furthermore, past research showed that certain brain diseases, such as Alzheimer's disease, epilepsy, Parkinson's, autism, and schizophrenia could be caused due to abnormal/absence neural synchronization^20,22,24–26^.

The synchronization of brain networks is essential for normal functioning, and understanding its dynamics is important to many aspects of our lives^27^. Research on synchronization is vital in revealing the role of communication cells in the neural network structure^28–30^. These communication cells are coupled by specialized intercellular pathways, which have been identified as gap junctions, and many researchers have been extensively studying the influence of the gap junctions on the synchronization behavior between two or three neuronal systems^28,31^ These gap junctions, known as protein channels, play an important role in the transmission of information between neurons. Many well-established mathematical models, such as the integrated-fire model (1907)^32^, the Hodgkin-Huxley model (HH)(1952)^33^, the FHN model (1962), the Morris and Lecar model (1981)^34^, and the Hind-marsh and Rose model (1984)^35^, have been used to study the synchronization of neurons. Among these mathematical models, the FHN neuron model, developed by Fitzhugh^36^ and Nagumo et al.^37^, has been used as a primary tool for investigating neuronal synchronization problems^20,28,38,39^. Different schemes, such as the backstepping design method^40^, adaptive synchronization method^41^, linear and nonlinear feedback synchronization method^28^, sliding mode control method^20^, time-delay feedback approach^42^, and impulsive synchronization method^43^, have been proposed to achieve synchronization in chaotic systems.

On the other hand, time delays are a fundamental part of almost all biological phenomena. The finite propagation speed of the action potential along the axons of neurons and time lapses in information transmission (synaptic process) and reception (dendritic process) between neurons produce time delays in real neurons and their networks^44^. Previous studies have shown that the speed of the action potential propagation could be significantly reduced (as slow as 1 m/s) by unmyelinated axons (myelin is an insulating layer that allows electrical impulses to transmit smoothly and quickly along the neuron) that cause large time delays (as high as 80 m/s) in information processing in neuronal networks^45,46^. Thus, time delays play an important role in the dynamics of neurons and their networks and should be included in mathematical modeling and analysis^28,47,48^. Recently, many researchers have investigated the effects of time delays in neuronal networks and found many delay-induced phenomena^49–52^. For instance, it has been reported that delay-enhanced synchronization may be essential for information transmission in neuronal networks^53,54^. It has also been reported that the firing dynamics of neurons are affected by many factors, such as noise, connection configuration, and coupling delays. For example, a recent study revealed that coupling delays present in the electrical or chemical synaptic connections can influence the synchronization of neuronal firing^55,56^.

Researchers have also studied the synchronization in time-delayed neuronal systems of two coupled neurons. Bin et al. used two coupled FHN neurons to examine the effects of time-delays on the synchronization dynamics between neurons^57^. Jia et al. investigated the influences and effects of time-delays on the dynamics of a coupled FHN neuronal system^58^. Wang et al. studied the synchronization phenomenon in the time-delayed chaotic system with unknown and uncertain parameters and developed an intermittent adaptive control scheme to guarantee synchronization between neurons^41^. Ibrahim and Jung developed an adaptive control methodology to guarantee synchronization between a bidirectional ring-structured FHN neuronal network under the effect of external electrical stimulation with single- and dual-state gap junctions^1^. Zhang investigated the out-lag synchronization by proposing a pinning control scheme in fractional-order complex networks with coupling and internal delays^59^. Petkoski and Jirsa studied how the phase lag synchronization between the neurons of different brain regions is governed by the spatio-temporal organization of the brain by using self-sustained time-delayed chaotic oscillators^60^. Liu and Puming recorded electrocorticogram (ECoG) data from eleven refractory epilepsy patients and utilized the phase synchronization phenomenon to map brain networks^61^. Siddique et al. used a chaotic master–slave system with unknown parameters, bounded delay rates, finite time-delay, and perturbations to investigate the synchronization phenomenon by developing local adaptive and robust adaptive control methodologies^62^. Siddique and Rehan proposed observer-based control strategies to realize synchronization in drive and response chaotic systems^63^. Jia et al. proposed a time-delayed coupled FHN system and studied its stability and Hopf bifurcation^58^. Riaz et al. used a nonlinear drive-response system with time-delay and under the effect of slope restricted input nonlinearity to investigate the synchronization phenomenon^64^. Zaheer et al. addressed the synchronization in non-linear coupled time-delayed systems by developing a novel state feedback delay-range-dependent control scheme^65^. Rehan and Hong studied the synchronization in coupled uncertain time-delayed FHN system with parametric variations under the effect of external electrical stimulation^66^.

Furthermore, the addition of noise in a dynamical system and its effects on the dynamical properties of the system are considered as one of the important research issues in the past decade, which has uncovered phenomena such as stochastic resonance^67^, coherent resonance^68^, and noise-sustained synchronization^69^ in nonlinear dynamical systems. Previous research has shown that neurons adjust their dynamics and firing behavior to transmit information optimally in the presence of noise^70^. Therefore, the addition of noise in time-delayed FHN neurons will make it more realistic but complex and difficult to investigate the synchronization in a network of neurons. However, as discussed above, a coupled system of two or three neurons cannot depict the dynamics of the network of neurons. Furthermore, the synchronization of noisy FHN neurons has not been investigated extensively in past research. Therefore, it is important to investigate and analyze the synchronization properties of a time-delayed network of FHN neurons in the absence and presence of noise. Ultimately, the development of control strategies for synchronization of a network of chaotic neurons with time delays, different direction-depend coupling (unidirectional and bidirectional), and noise, particularly under external disturbances, is very important and challenging.

**Figure 1:**
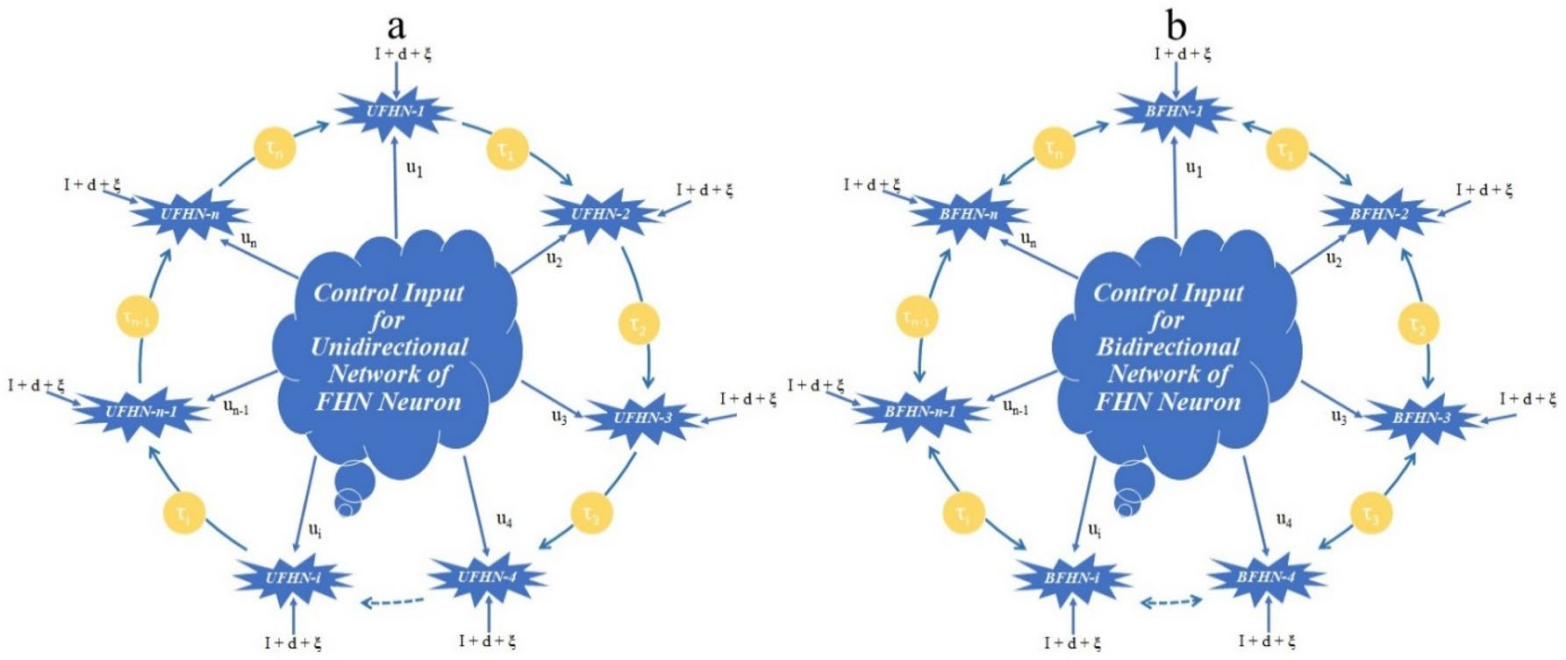
Schematic diagram of ring-structured delayed FHN neuronal networks. (**a**) Unidirectional coupling. (**b**) Bidirectional coupling.

This paper examines the synchronization phenomena of unidirectional and bidirectional ring-structured FHN networks with and without noise under ionic and EES with different gap junctions and time-delay dynamics between the neurons. Each neuron of the unidirectional network is connected to the next neuron via synapses, and the first neuron of the network is a master neuron for connecting networks. However, each neuron in a bidirectional network is connected to the next neuron as well as the previous neuron behaves as a master and slave neuron simultaneously. The configuration of both networks is shown in Fig. 1a, b. The separation between each neuron in both networks is incorporated through different time-delay parameters.

This paper also investigates the influence of the time delay between connected neurons on synchronization conditions. Unique and different adaptive control laws are proposed for both unidirectional and bidirectional neuronal networks, which guarantee the synchronization of time-delayed FHN networks in the absence and presence of noise. Necessary and sufficient conditions were driven using the Lyapunov theory of stability, which also assures the synchronization of delayed FHN networks. Furthermore, the proposed control schemes were verified using multiple networks consisting of five, ten, fifty, one hundred, two hundred and fifty, five hundred, and one thousand time-delayed FHN neurons with and without noise through numerical simulations.

The main contributions of this paper include (1) investigating the synchronization of a unidirectional network of *n-*FHN neurons in the absence and presence of noise with different gap-junctions and time delays, (2) investigating the synchronization of a bidirectional network of *n-*FHN neurons in the absence and presence of noise with different gap-junctions and time delays, (3) the development of unique and different adaptive control laws for both unidirectional and bidirectional networks, and (4) achieving the synchronization of membrane and recovery states for both unidirectional and bidirectional FHN neurons using the proposed adaptive schemes.

### Time-delayed FHN neuronal networks and control design

This section presents the mathematical formulation of the synchronization problem for unidirectional and bidirectional neuronal networks of *n* time-delayed FHN neurons with and without noise connected in a ring structure, under ionic and EES, and with different time delays and gap junctions. Based on the literature, we hypothesized that both unidirectional and bidirectional time-delayed FHN neuronal networks will show very complex and unpredictable behaviors and dynamics with delays in gap junctions. Furthermore, the inclusion of the noise and dynamic effects of ionic gate disturbances in the network will make it more realistic but more challenging to analyze.

#### Definition

A system of neurons is said to be synchronized if for all initial conditions $x_{i} (0)$ and $y_{i} (0)$, $\mathop {\lim }\limits_{x \to \infty } \left\| {e_{{x_{i} }} } \right\| = 0$ and $\mathop {\lim }\limits_{y \to \infty } \left\| {e_{{y_{i} }} } \right\| = 0$.

### Unidirectional time-delayed FHN neuronal networks

- **Network without noise**

Let us consider a unidirectional ring-structured FHN network with different gap-junctions, time delays, and without any external noise between the synapses of connecting neurons. Mathematically,

1 $$\begin{aligned} \dot{x}_{i} & = x_{i} (x_{i} - 1)(1 - rx_{i} ) - y_{i} - g_{i} (x_{i} (t - \tau_{i} ) - x_{i + 1} ) + I + d + u_{i} \\ \dot{y}_{i} & = bx_{i} - cy_{i} \\ \end{aligned}$$

where $x_{i}$ and $y_{i}$ are stated action potential and recovery variables, respectively; $r$, $b$, and $c$ are positive constants; $d = 0.01\sin (0.2t)$ is the ionic gate disturbance;$I = \frac{A}{\omega }\cos (\omega t)$ is the stimulus current; $g_{i}$ represents the coupling strength of the gap junction between master and slave neurons; $\tau_{i}$ is non-negative delay parameter; $u_{i}$ represents the control; and $i = 1,2 \ldots ,n$ is the number of FHN neurons.

(b) **Network with noise**

Let us consider a unidirectional ring-structured FHN network with different gap-junctions, time delays, and external noise between the synapses of connecting neurons. Mathematically,

2 $$\begin{aligned} \dot{x}_{i} & = x_{i} \left( {x_{i} - 1} \right)\left( {1 - rx_{i} } \right) - y_{i} - g_{i} \left( {x_{i} (t - \tau_{i} ) - x_{i + 1} } \right) + I + d + \varphi_{1} + u_{i} \\ \dot{y}_{i} & = bx_{i} - cy_{i} \\ \end{aligned}$$

where $\varphi_{1} (t)$ is a Gaussian noise^70^ source having zero mean and correlation function:

3 $$\langle \varphi_{1} (t)\varphi_{1} (t^{\prime})\rangle = 2D\delta (t - t^{\prime}).$$

(c) **Control laws design**

The error states of the systems described by Eqs. (1) and (2) can be defined as

4 $$e_{{x_{i} }} = x_{i} (t - \tau_{i} ) - x_{i + 1} ,\,\,e_{{y_{i} }} = y_{i}(t - \tau_{i} ) - y_{i + 1}$$

Taking the time derivative of the error system, Eq. (4) can be expressed as shown in Eq. (5).

5 $$\begin{aligned} \dot{e}_{{x_{i} }} & = - re_{{x_{i} }} \left( {x_{i}^{2} \left( {t - \tau_{i} } \right) + x_{i} \left( {t - \tau_{i} } \right)x_{i + 1} + x_{i + 1}^{2} } \right) + \left( {re_{{x_{i} }} + e_{{x_{i} }} } \right)\left( {x_{i} \left( {t - \tau_{i} } \right) + x_{i + 1} } \right) - e_{{x_{i} }} \\ & \quad - e_{{y_{i} }} - g_{i} \left( {x_{i} \left( {t - 2\tau_{i} } \right) - x_{i + 1} \left( {t - \tau_{i} } \right) } \right) + g_{i + 1} \left( {x_{i + 1} \left( {t - \tau_{i + 1} } \right) - x_{i + 2} } \right) + u_{i} - u_{i + 1} \\ \dot{e}_{{y_{i} }} & = b\left( {x_{i} \left( {t - \tau_{i} } \right) - x_{i + 1} } \right) - ce_{{y_{i} }} \\ \end{aligned}$$

Next, we propose a unique control scheme by using Lyapunov stability and adaptive control theories.

#### Theorem 1

*Consider the unidirectional time-delayed FHN systems as described in* *Eqs.* (1) and (2) *with the dynamical error system described by* Eq. (5). *If the controllers* $u_{i}$ *in the system defined by* Eq. (5) *are defined as*

$$u_{i} = (b + c)\left[ {\left( {x_{i - 1} \left( {t - \tau_{i-1} } \right) - x_{i} } \right)\left( {\exp \left( {\left( {x_{i} \left( {t - \tau_{1} } \right)} \right) + \left( {x_{i + 1} \left( {t - \tau_{1} } \right) + 1} \right)} \right)} \right)} \right],$$

*then the synchronization of unidirectional networks of the time-delayed FHN systems described in Eqs.* (1) *and* (2) *can be achieved by converging the error of the synchronized system to zero.*

#### *Proof*

Please see the supplementary information for the proof.

### Bidirectional time-delayed FHN neuronal networks

- **Network without noise**

Let us consider a bidirectional ring-structured time-delayed FHN network with different gap junctions and without any external noise. Mathematically,

6 $$\begin{aligned} \dot{x}_{j} & = x_{j} (x_{j} - 1)(1 - rx_{j} ) - y_{j} - g_{2j - 1} (x_{j} - x_{j - 1}(t - \tau_{j - 1} ) ) - g_{2j} (x_{j} (t - \tau_{j} ) - x_{j + 1} ) \\ & \quad + I + d + u_{j} \\ \dot{y}_{j} & = bx_{j} - cy_{j} \\ \end{aligned}$$

where $g_{2j - 1}$ and $g_{2j}$ represent the coupling strength of the gap junction between the master and slave neurons, and $\tau_{j - 1}$ and $\tau_{j}$ are the delay parameters between the neurons, for *j* = 1, 2, 3, …, *n*.

(b) **Network with noise**

Let us consider a bidirectional ring-structured time-delayed FHN network with different gap junctions and external noise. Mathematically,

7 $$\begin{aligned} \dot{x}_{j} & = x_{j} \left( {x_{j} - 1} \right)\left( {1 - rx_{j} } \right) - y_{j} - g_{2j - 1} (x_{j} - x_{j - 1}(t - \tau_{j - 1} ) ) - g_{2j} (x_{j} (t - \tau_{j} ) - x_{j + 1} ) \\ & \quad + I + d + \varphi_{2} + u_{j} \\ \dot{y}_{j} & = bx_{j} - cy_{j} \\ \end{aligned}$$

where $\varphi_{2} (t)$ is the Gaussian noise^70^ source having zero mean and the following correlation function:

8 $$\langle \varphi_{2} (t)\varphi_{2} (t^{\prime})\rangle = 2D\delta (t - t^{\prime}).$$

(c) **Control laws design**

Taking the derivative of Eq. (4) with respect to time, the error system for the time-delayed FHN networks described in Eqs. (6) and (7) can be expressed as shown by Eq. (9).

9 $$\begin{aligned} \dot{e}_{{x_{j} }} & = - re_{{x_{j} }} \left( {x_{j}^{2} \left( {t - \tau_{j} } \right) + x_{j} \left( {t - \tau_{j} } \right)x_{j + 1} + x_{j + 1}^{2} } \right) + \left( {re_{{x_{j} }} + e_{{x_{j} }} } \right)\left( {x_{j} \left( {t - \tau_{j} } \right) + x_{j + 1} } \right) - e_{{x_{j} }} \\ & \quad - e_{{y_{j} }} - g_{2j - 1} \left( {x_{j} \left( {t - \tau_{j} } \right) - x_{j - 1} \left( {t - \tau_{j}- \tau_{j-1} } \right) } \right) - g_{2j} e_{{x_{j}}} \left( {t - \tau_{j} } \right) + g_{2j + 1} \left( {x_{j + 1} - x_{j} \left( {t - \tau_{i} } \right)} \right) - g_{2j + 2} e_{{x_{j + 1} }} \\ & \quad + u_{j} - u_{j + 1} \\ \dot{e}_{{y_{j} }} & = b\left( {x_{j} \left( {t - \tau_{j} } \right) - x_{j + 1} } \right) - ce_{{y_{j} }} \\ \end{aligned}$$

Next, we propose a unique control scheme by using Lyapunov stability and adaptive control theories.

#### Theorem 2

*Consider bidirectional time-delayed FHN networks as described in Eqs.* (6) and (7) *with the dynamical error system of* Eq. (9). *If the controllers* $u_{j}$ *in the error system are defined as*

$$u_{j} = (b + c)\left[ {\left( {x_{j - 1} \left( {t - \tau_{j-1} } \right) - x_{j} } \right)\left( {\exp \left( {\left( {x_{j} \left( {t - \tau_{1} } \right)} \right)\left( {x_{j + 1} \left( {t - \tau_{1} } \right) + 1} \right)} \right)} \right)} \right] + \left( {x_{j - 1} \left( {t - \tau_{j-1} } \right) - x_{j} } \right)$$

*then this will ensure the synchronization of the bidirectional networks of the time-delayed FHN systems described in Eqs.* (6) *and* (7) *by converging the error of the synchronized system to zero*.

#### *Proof*

Please see the supplementary information for the proof.

## Results

After creating an accurate model for the dynamics of the ring-structured network of *n*-identical FHN neurons with and without noise, we establish a synchronization control scheme for achieving the coherent behavior of the neurons. Numerical simulations were executed to verify the proposed control laws and examine their impact on the synchronization of time-delayed unidirectional and bidirectional ring-structured FHN networks composed of five, ten, fifty, one hundred, two hundred and fifty, five hundred, and one thousand neurons. The parameter values used in this study are listed in Table 1. The values for *g*, $\tau$, and initial conditions are randomly chosen from (0–0.1), (3–35), and ((0–0.5), (0–0.5)), respectively.

**Table 1:** Values of the parameters used in the numerical simulations.

Parameter	Value
*r*	10
*b*	1
*c*	0.003
*A*	0.1
*w*	2π*f*
*f*	0.131

### Analysis of unidirectional time-delayed FHN networks

- **Networks without noise**

The proposed unidirectional networks of five, ten, fifty, one hundred, two hundred and fifty, five hundred, and one thousand neurons with different gap-junctions and time delays can be modeled as presented in Eq. (1) with $i = 1,2, \ldots ,1000$**.**

**Figure 2:**
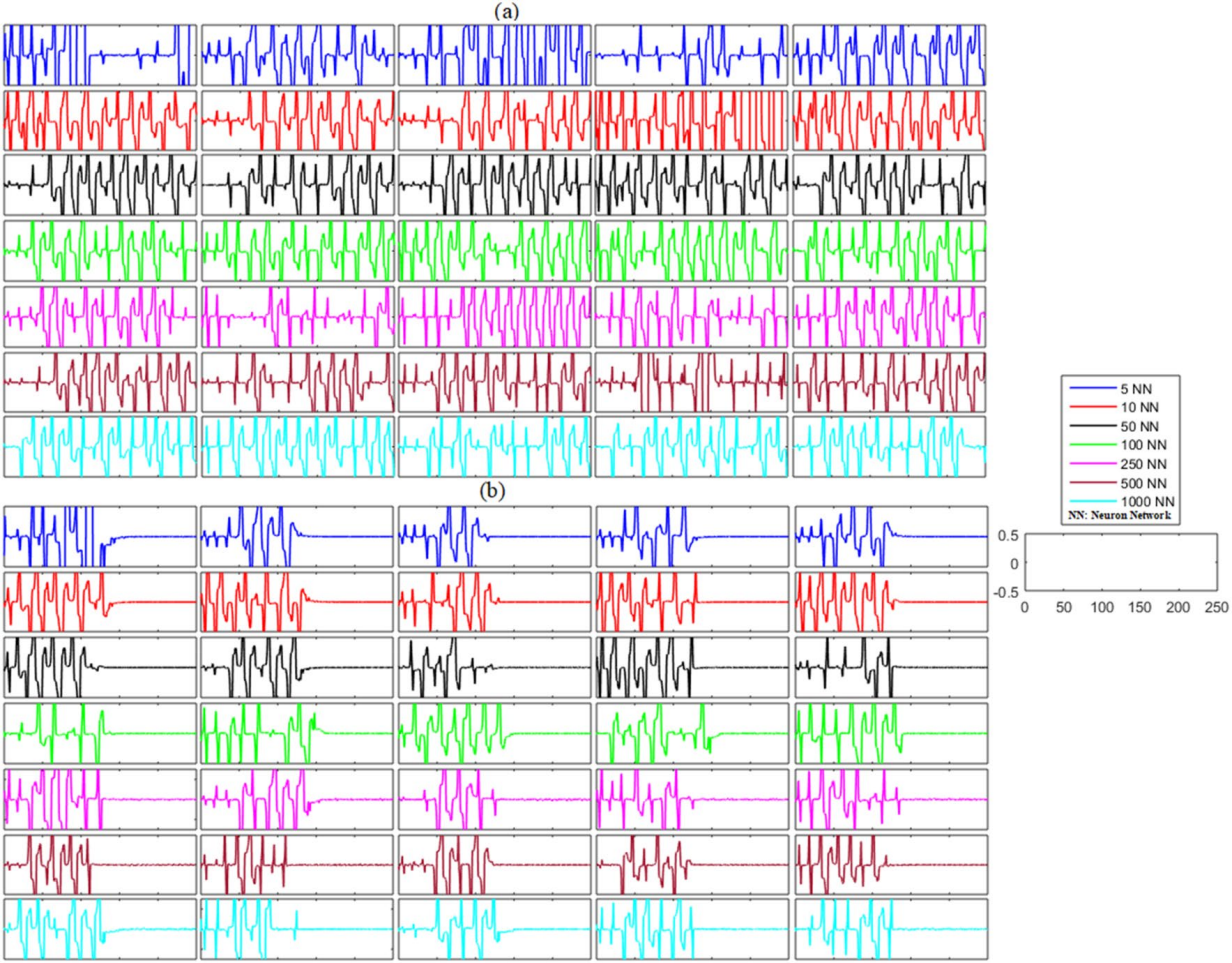
(**a**) Without control synchronization dynamics of the membrane potential states (x-states) of different time-delayed unidirectional FHN networks without noise. (**b**) Controlled synchronized error dynamics of the membrane potential states (x-state) of different time-delayed unidirectional FHN networks without noise.

**Figure 3:**
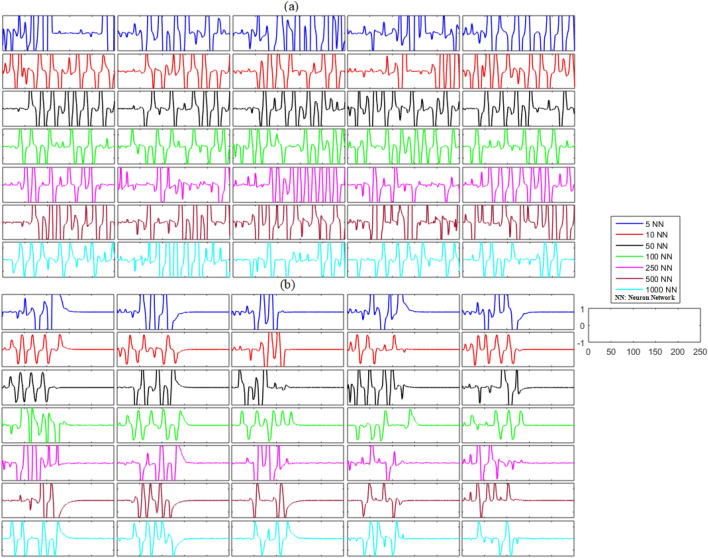
(**a**) Without control synchronization dynamics of the recovery variable states (y-states) of different time-delayed unidirectional FHN networks without noise. (**b**) Controlled synchronized error dynamics of the recovery variable states (y-states) of different time-delayed unidirectional FHN neuronal networks without noise.

In the case of the unidirectional network without any noise, the unsynchronized error dynamics for the membrane potential states and recovery variable states for the network of five neurons (blue line), ten neurons (red line), fifty neurons (black line), one hundred neurons (green line), two hundred and fifty neurons (magenta line), five hundred neurons (brown line), and one thousand neurons (cyan line) are shown in Figs. 2a and 3a. These figures show the error dynamics of five randomly selected pairs of neurons from each network. These plots reveal that the error dynamics are not convergent for both states and the activity of neurons in all networks is highly non-synchronized.

Next, we analyzed the results shown in Figs. 2b and 3b after the implementation of the proposed control laws for the unidirectional time-delayed network without noise. We analyzed the effectiveness of the proposed control scheme by examining the synchronization dynamics of the ring-structured delayed FHN neuronal network, with and without the adaptive control. The outcomes of this examination are shown in Figs. 2b and 3b. The proposed controllers for the unidirectional network were activated at *t* = 130. The results show oscillatory and non-synchronized behavior for all networks before the application of the proposed controller. In contrast, when the controller was activated at *t* = 130, the errors between the membrane potential and recovery variables converged to zero, indicating the synchronization between both states of the delayed unidirectional FHN neuronal networks. Furthermore, we calculated the mean errors for both membrane potential states and recovery variable states in each network to show the overall effectiveness of the proposed control scheme for the non-noisy unidirectional network of time-delayed FHN networks. The results of this analysis are listed in Table 2. It can be visualized that the error for both states in each network is almost zero, indicating the synchronized behavior of the network.

(b) **Networks with noise**

**Table 2:** Mean errors in the synchronization dynamics of the membrane potential states and recovery variable states for different-sized unidirectional networks of FHN neurons without noise.

Number of neurons in network	Membrane potential states	Recovery variable states
5	− 1.2734 × 10^−21^	0
10	− 1.0508 × 10^−20^	3.0227 × 10^−20^
50	− 1.4520 × 10^23^	− 3.2524 × 10^−21^
100	6.8429 × 10^−22^	4.6108 × 10^−22^
250	2.9745 × 10^−21^	1.0913 × 10^−20^
500	8.0480 × 10^−23^	0
1000	− 5.3321 × 10^−23^	− 9.0176 × 10^−22^

**Figure 4:**
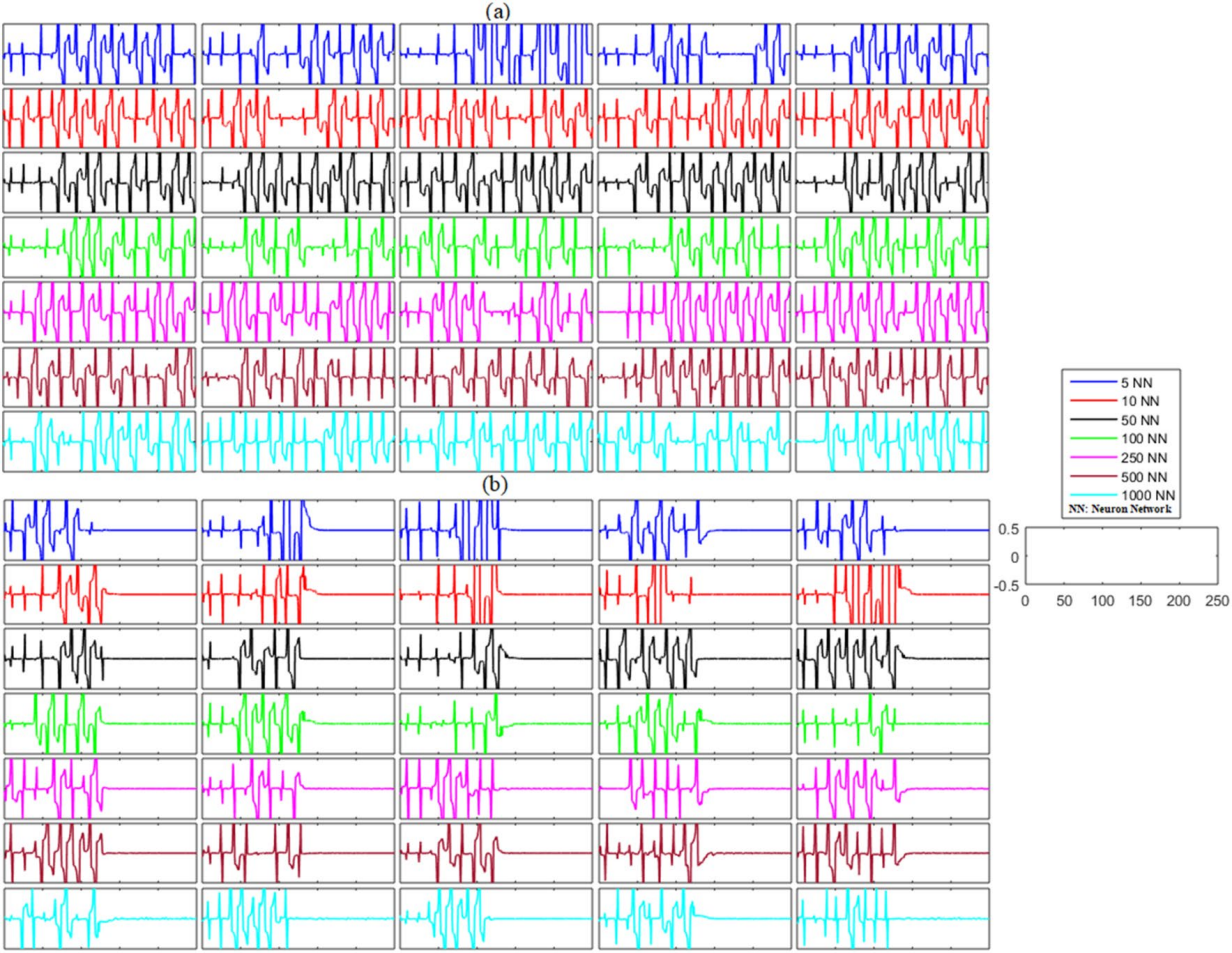
(**a**) Without control synchronization dynamics of the membrane potential states (x-states) of different time-delayed unidirectional FHN networks with noise. (**b**) Controlled synchronized error dynamics of the membrane potential states (x-state) of different time-delayed unidirectional FHN networks with noise.

**Figure 5:**
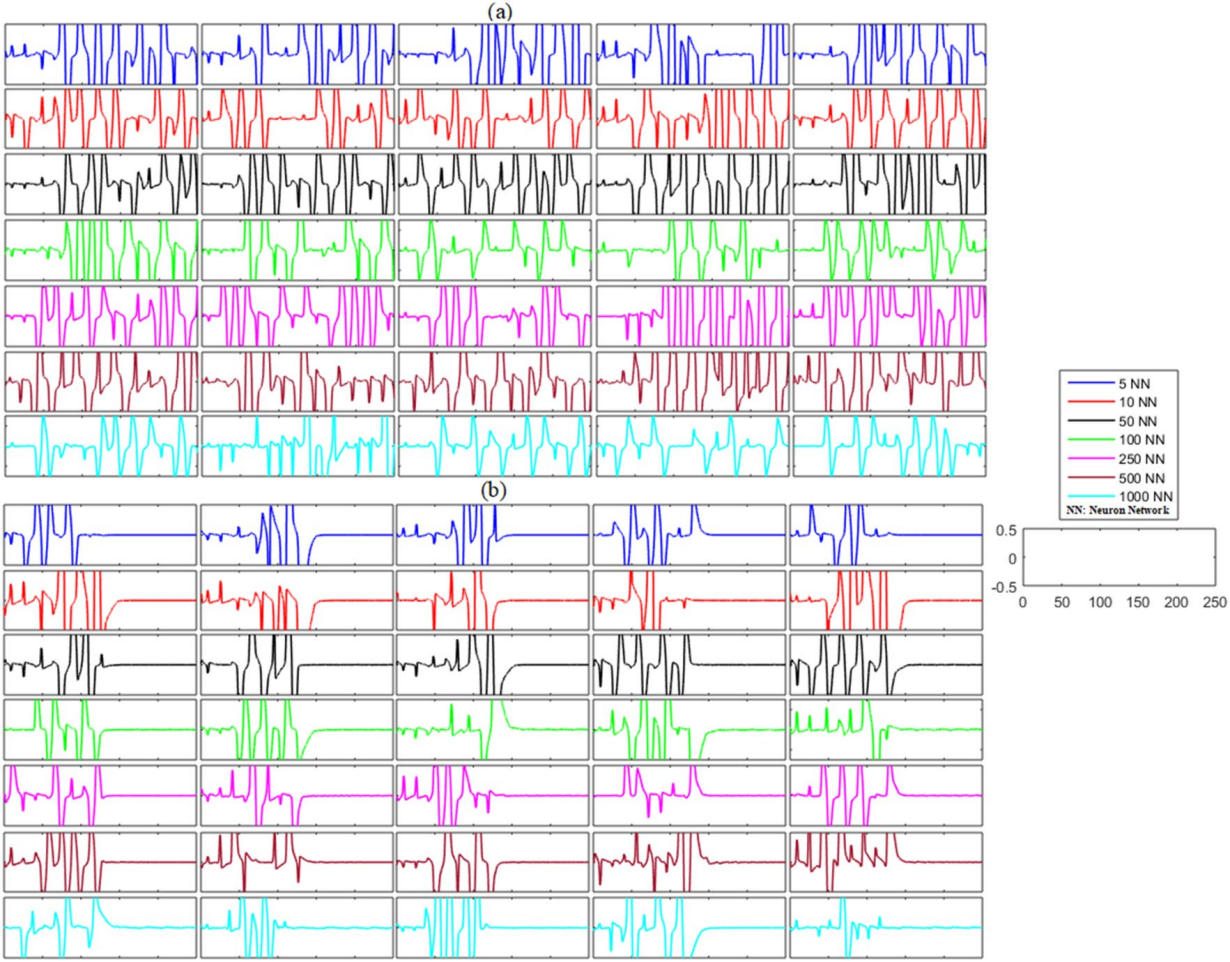
(**a**) Without control synchronization dynamics of the recovery variable states (y-states) of different time-delayed unidirectional FHN networks with noise. (**b**) Controlled synchronized error dynamics of the recovery variable states (y-states) of different time-delayed unidirectional FHN neuronal networks with noise.

Next, we investigated the synchronization problem in unidirectional networks with noise. The results of this analysis are shown in Figs. 4 and 5 and Table 3. The results in Figs. 4a and 5a show the unexpected behavior of the neurons in each network and indicate that the primary neuronal networks of delayed unidirectional FHN neurons with noise are not synchronized. It can be concluded at this stage that the non-synchronized behavior is present during the firing of the unidirectional neurons of the delayed networks. The results in Figs. 4b and 5b illustrate the effectiveness of the proposed control scheme for noisy networks of FHN neurons. Initially, the controller was switched off until *t* = 130, and it can be seen that five randomly selected error dynamics show non-zero and non-convergent spikey behavior, indicating that the activity of the neurons in each network is non-synchronized. In contrast, the error dynamics converged to zero and all the networks achieved synchronization as soon as the controller is switched on at *t* = 130. Furthermore, the mean errors for each state and each network—listed in Table 3—show that the proposed control scheme is successful in guaranteeing the synchronization of noisy FHN neuronal networks regardless of the size of the network.

**Table 3:** Mean errors in the synchronization dynamics of the membrane potential states and recovery variable states for different-sized unidirectional networks of FHN neurons with noise.

Number of neurons in network	Membrane potential states	Recovery variable states
5	− 2.0014 × 10^−21^	8.7445 × 10^−20^
10	1.6192 × 10^−21^	− 2.0768 × 10^−20^
50	4.5356 × 10^−22^	− 1.5868 × 10^−20^
100	− 1.0464 × 10^−22^	1.0685 × 10^−22^
250	2.1871 × 10^−22^	− 1.0531 × 10^−21^
500	− 7.8048 × 10^−22^	− 1.7182 × 10^−22^
1000	5.3821 × 10^−24^	− 4.8910 × 10^−22^

### Analysis of bidirectional time-delayed FHN networks

- **Networks without noise**

**Figure 6:**
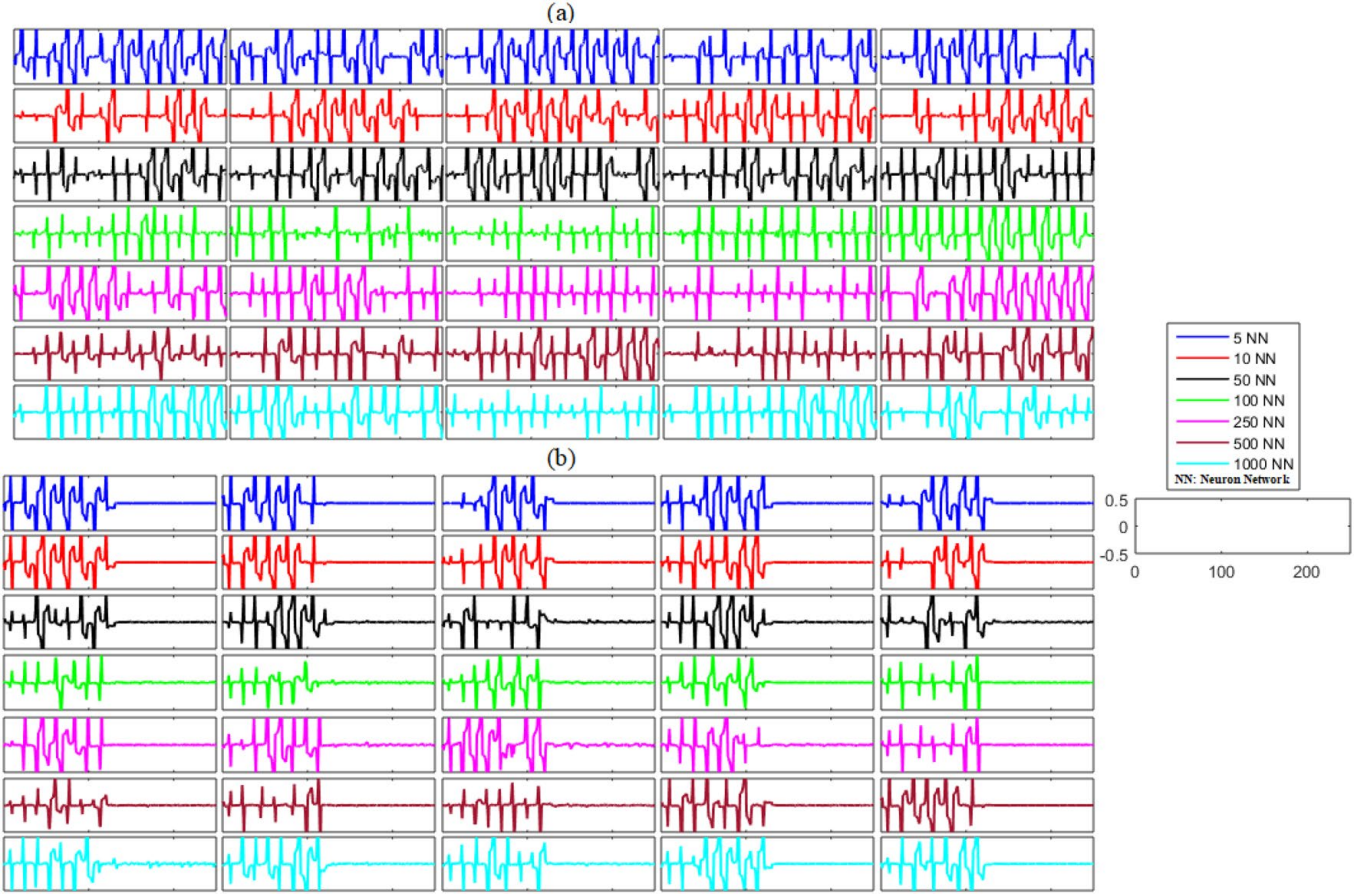
(**a**) Without control synchronization dynamics of the membrane potential states (x-states) of different time-delayed bidirectional FHN networks without noise. (**b**) Controlled synchronized error dynamics of the membrane potential states (x-state) of different time-delayed bidirectional FHN networks without noise.

**Figure 7:**
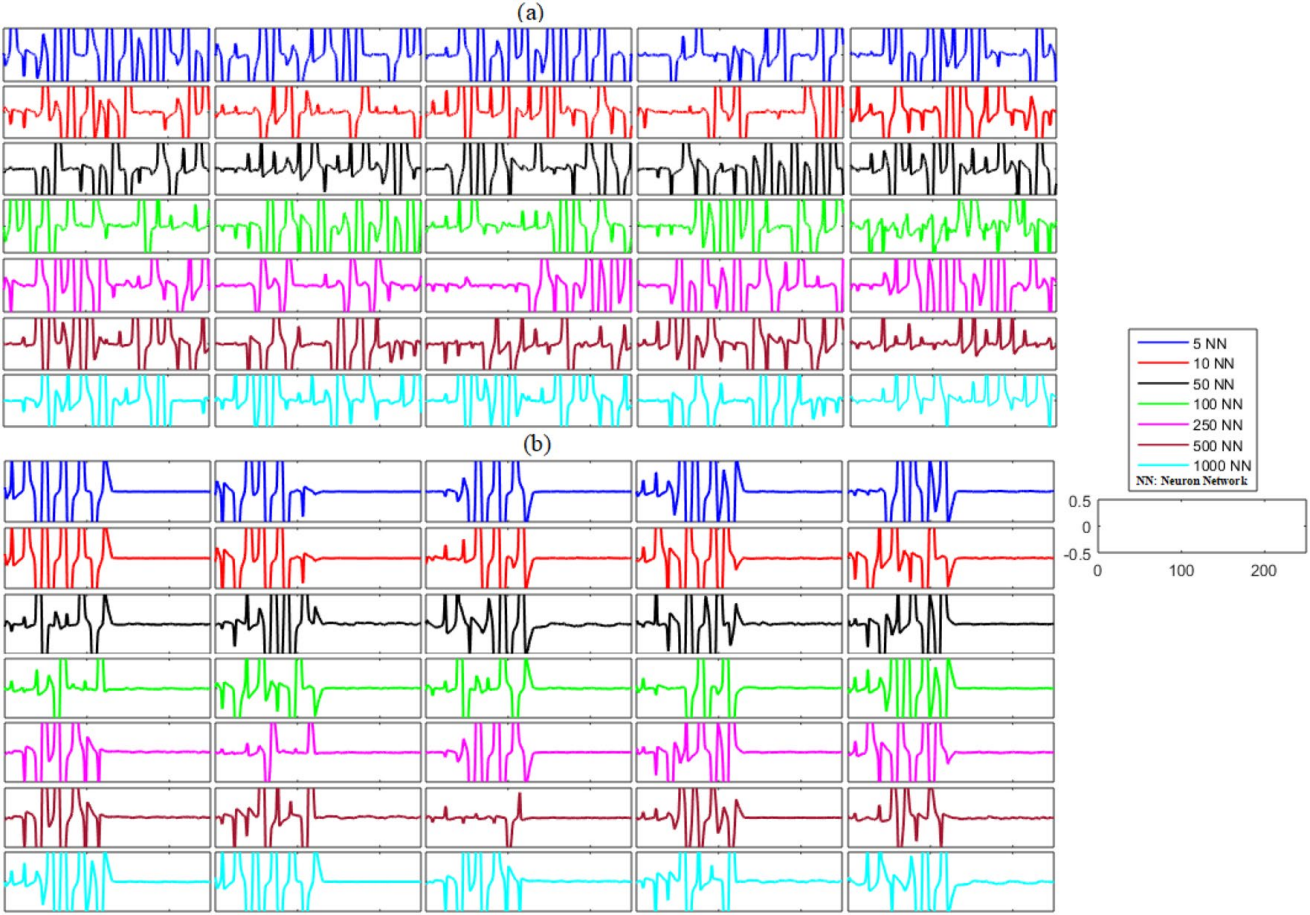
(**a**) Without control synchronization dynamics of the recovery variable states (y-states) of different time-delayed bidirectional FHN networks without noise. (**b**) Controlled synchronized error dynamics of the recovery variable states (y-states) of different time-delayed bidirectional FHN neuronal networks without noise.

Similar to the unidirectional network’s analysis, we considered bidirectional networks of five, ten, fifty, one hundred, two hundred and fifty, five hundred, and one thousand neurons with different gap-junctions and time delays. The results of this analysis are illustrated in Figs. 6 and 7 and Table 4. The non-synchronized activity of neurons in different bidirectional networks without noise can be visualized in Figs. 6a and 7a as the time dynamics of the errors are non-zero and non-convergent for both membrane potential states and recovery variable states. Contrastingly, both states of the networks achieved zero error after the application of the proposed control scheme at *t* = 130, as shown in Figs. 6b and 7b. The mean errors for both states of each network are listed in Table 4. These results suggest that each state of the delayed neuronal network achieved synchronized behavior with the state of the corresponding slave neuron because the outcome of the time dynamics of error converged to zero.

(b) **Networks with noise**

**Table 4:** Mean errors in the synchronization dynamics of the membrane potential states and recovery variable states for different-sized bidirectional networks of FHN neurons without noise.

Number of neurons in network	Membrane potential states	Recovery variable states
5	8.9592 × 10^−22^	0
10	5.1245 × 10^−20^	1.7956 × 10^−19^
50	− 3.8708 × 10^−20^	4.8477 × 10^−21^
100	2.6293 × 10^−20^	9.3919 × 10^−21^
250	− 1.8942 × 10^−21^	4.0780 × 10^−21^
500	− 3.5844 × 10^−21^	2.0709 × 10^−20^
1000	1.5764 × 10^−21^	− 1.0755 × 10^−21^

**Figure 8:**
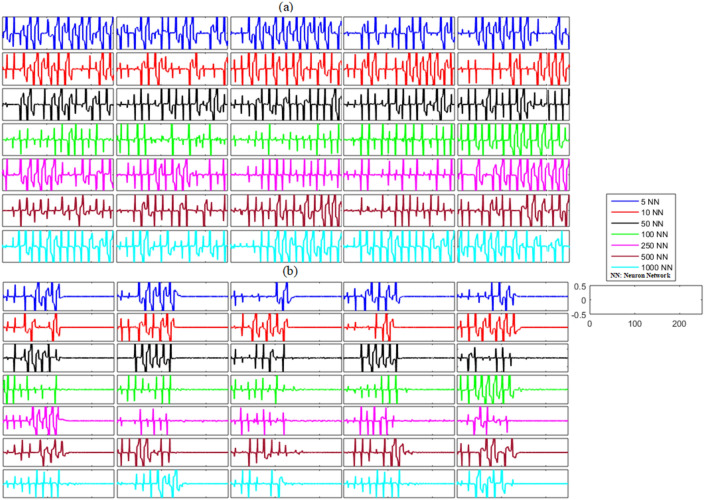
(**a**) Without control synchronization dynamics of the membrane potential states (x-states) of different time-delayed bidirectional FHN networks with noise. (**b**) Controlled synchronized error dynamics of the membrane potential states (x-state) of different time-delayed bidirectional FHN networks with noise.

**Figure 9:**
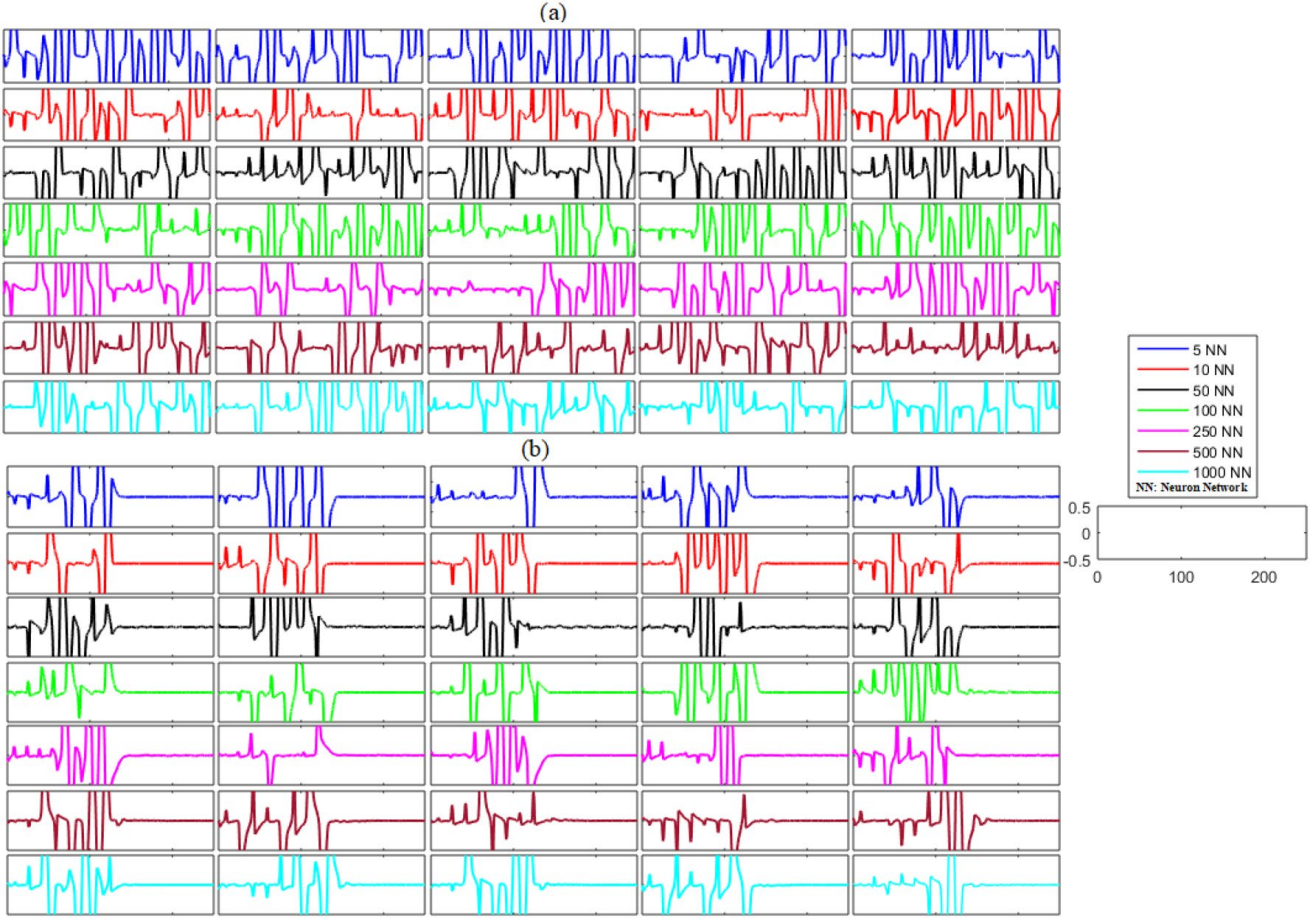
(**a**) Without control synchronization dynamics of the recovery variable states (y-states) of different time-delayed bidirectional FHN networks with noise. (**b**) Controlled synchronized error dynamics of the recovery variable states (y-states) of different time-delayed bidirectional FHN neuronal networks with noise.

The dynamical structure of the bidirectional delayed network with noise is more complex and complicated than that of the unidirectional delayed FHN neuronal networks, but the results of the numerical simulations suggest that synchronization can be achieved successfully with the activation of the proposed controller at *t* = 130. The time–error dynamics of different-sized networks revealed in Figs. 8a and 9a show the unsynchronized neuronal activities between all neurons for all networks. Next, we analyzed the results after the implementation of the proposed control law for the bidirectional delayed network in Figs. 8b and 9b. These results show the error dynamics before and after the application of the proposed adaptive controller. Similar to the previous cases, the delayed networks showed highly non-synchronized behavior until the proposed controller was switched on. Then, as soon as the controller was applied, all the errors converged to zero, indicating the effectiveness of the proposed scheme. Furthermore, the results listed in Table 5 with very low mean errors also show that different bidirectional networks of noisy time-delayed FHN neurons achieved synchronization, indicating the effectiveness of the proposed scheme.

**Table 5:** Mean errors in the synchronization dynamics of the membrane potential states and recovery variable states for different-sized bidirectional networks of FHN neurons with noise.

Number of neurons in network	Membrane potential states	Recovery variable states
5	− 4.9363 × 10^−21^	− 1.1989 × 10^−20^
10	− 5.0001 × 10^−21^	− 3.4012 × 10^−20^
50	1.3890 × 10^−21^	4.0569 × 10^−21^
100	7.9239 × 10^−22^	2.6784 × 10^−21^
250	− 5.9572 × 10^−22^	− 1.0239 × 10^−22^
500	− 1.2124 × 10^−21^	− 5.6866 × 10^−22^
1000	− 2.7764 × 10^−21^	2.0092 × 10^−21^

The convergence of the error dynamics to zero in all cases guaranteed the synchronization of the different-sized ring-structured networks of FHN neurons in the absence and presence of noise with different gap-junctions and time delays under conditions of EES and ionic gate disturbance. It was also observed that the errors between neurons for unidirectional and bidirectional gap-junction networks converged to zero very rapidly in all cases, showing the efficiency of the proposed control schemes.

## Discussion

The processing of cognitive information in the brain is based upon the synchronized interactions between large numbers of neurons distributed within and across different specialized brain regions. Experimental and theoretical results from previous studies suggested that synchronization of neuronal activity is not only a fundamental property of cortical and subcortical networks within and across different brain regions but also serves a variety of functions in cognitive processes. It is evident from past research that certain brain disorders, such as Alzheimer's disease, epilepsy, Parkinson's, autism, and schizophrenia are associated with abnormal neural synchronization^19^. Researchers have studied the synchronization problem in neurons by using different mathematical models of neurons^71–73^. Among those, FHN is the most commonly used model to investigate the synchronization of coupled neurons because of its wide applicability and complex dynamical aspects. In the literature, the subject of neuronal synchronization, using the FHN model, has been intensively examined as a potential application in cognitive engineering^1,20,28,47,57^. Researchers have developed adaptive^20,41^, nonlinear^28^, robust control^23^, neural-network-, fuzzy^74^, and observer-based control schemes^63^ to study the synchronization phenomenon in FHN neurons under external electrical stimulations. However, these conventional methodologies were developed for two or three coupled neurons and cannot guarantee synchronization of distant neurons if used for synchronizing the activity of networks of neurons because the mathematical models ignore the time delays arising from the separation between coupled neurons, and hence cannot synchronize distant FHN neurons. Therefore, the addition of a delay term in mathematical models accounting for the distant communication between neurons makes it realistic but more complex to study the synchronization problem. Furthermore, the integration of the gap-junction strength in FHN neurons renders the synchronization dilemma nontrivial^75^. Moreover, previous studies assumed bidirectional coupling between the neurons whereas experimental observation suggests that the coupling between neurons could be unidirectional and may result in different coupling strengths for each neuron^75,76^. Therefore, the dynamical effects of unidirectional gap junctions while entertaining time delays (due to neuronal separation) in the mathematical models should not be ignored. Additionally, it is evident from past research that the presence of noise can affect the dynamical behavior of the neuronal system and neurons adjust their firing properties to transmit information optimally^70^. Therefore, researchers should entertain the noise effect in their mathematical models to study the synchronization problem.

To overcome the shortcomings of previous studies, this study investigated the synchronization problem in the network of coupled FHN neurons by incorporating time delays, different direction-dependent coupling (unidirectional and bidirectional), noise, and ionic and external disturbances in mathematical models. In contrast to traditional techniques, we proposed simple adaptive control schemes that guarantee the synchronization of unidirectional and bidirectional time-delayed networks of FHN neurons in the absence and presence of noise. The efficacy of the proposed control laws is shown through numerical simulations of different-sized networks consisting of five, ten, fifty, one hundred, two hundred and fifty, five hundred, and one thousand neurons. The results presented in Figs. 2, 3, 4, 5, 6, 7, 8 and 9 and Tables 2, 3, 4 and 5 validate the efficiency of the proposed control laws.

Although the present study proposed efficient control schemes that guarantee the synchronization of networks of time-delayed FHN neurons, it also has some limitations/drawbacks. One obvious of this study is that we considered the networks of identical FHN neurons with known fixed parameters in the present configurations. However, the possibility of two coupled neurons to be non-identical cannot be ignored, and the actual model parameters cannot be completely known, owing to the known dynamics of the brain and biological restrictions. Therefore, considering a network of large numbers of non-identical neurons having unknown parametric values where several hundred neurons communicate with each other under different direction-dependent couplings (unidirectional and bidirectional), can enhance the complexity and analysis of neuronal synchronization. The understanding of the physical theory behind direction-dependent couplings, it's discrete modeling, and simulations is a complex and tedious job; hence, it is a challenging task for the research community. Thus, it is our immediate future plan to study the synchronization of the network of non-identical FHN neurons with unknown parameters and disturbances. Moreover, in the current configurations, the coupling between the neurons of each network is assumed to be unidirectional or bidirectional, but in a real scenario, a network may have both types of direction-dependent coupling that will enhance the complexity of the network. Another possible future direction of the current study is that we could investigate the link between mathematical simulations and experimental data recorded from real neurons, as this could help to understand the dynamics of various brain disorders^19^. This could be done by estimating the parameters of FHN neurons to replicate the experimental data. For instance, Che et al.^77^ developed an identification method to estimate the parameters of FHN models to replicate the experimental data recorded from real neurons. Furthermore, in the present study, only synchronization within a network of neurons is considered and how two or more networks with different dynamics and configurations (non-identical neurons, unknown parameters, external disturbances, and different direction-dependent coupling) communicate and synchronize their activities is yet to be explored in future work.

## Conclusion

The development of control strategies for synchronization of a network of chaotic neurons with time delays, different direction-dependent coupling (unidirectional and bidirectional), and noise, particularly under external disturbances, is essential and very challenging. Most of the previous studies developed control strategies for two or three coupled neurons with bidirectional coupling and without incorporating the effect of noise, but not for time-delayed neural networks. To overcome these limitations, this study investigated the synchronization problem in the network of coupled FHN neurons by incorporating time delays, different direction-dependent coupling (unidirectional and bidirectional), noise, and ionic and external disturbances in mathematical models. Both unidirectional and bidirectional time-delayed FHN neuronal networks have very complex and unpredictable behavior and dynamics. In this study, the lag synchronization of a network of delayed FHN neurons with unidirectional and bidirectional coupling in the absence and presence of noise was addressed. Different gap junctions and time-delay parameters were used to incorporate the dynamics of time delays in neurons. Two different networks, one with unidirectional coupling between two neurons and the other with bidirectional coupling in membrane states, were considered. To achieve the synchronization between the states of the delayed neuronal networks, we designed two different adaptive control strategies, which compensated for the nonlinear dynamics without direct cancelation. Lyapunov stability theory was used to derive sufficient conditions that guarantee the synchronization of the delayed FHN neuronal networks. Numerical simulations with networks of five, ten, fifty, one hundred, two hundred and fifty, five hundred, and one thousand neurons were performed to demonstrate the efficiency of the proposed control schemes.

## Supplementary Information

Supplementary Information.
