# Supplementary material for: Lag synchronization of coupled time-delayed FitzHugh–Nagumo neural networks via feedback control
Source: Sci Rep. 2021 Feb 16;11:3884. doi: 10.1038/s41598-021-82886-x (PMC7887243; doi:10.1038/s41598-021-82886-x)
Supplement: Supplementary file 1 — Supplementary Information. [file 41598_2021_82886_MOESM1_ESM.docx]

**Supplementary Information**

**Lag synchronization of coupled time-delayed FitzHugh-Nagumo neural networks via feedback control**

Malik Muhammad Ibrahim ^1^, Muhammad Ahmad Kamran ^2^, Malik Muhammad Naeem Mannan ^3^, Il Hyo Jung ^1, *^, and Sangil Kim ^1^, *

1. Department of Mathematics, Pusan National University, Busan 46241, Republic of Korea.
2. Department of Cogno-Mechatronics Engineering, Pusan National University, Busan 46241, Republic of Korea.
3. School of Allied Health Sciences, Griffith University, Gold Coast, QLD 4222 Australia.

***** Correspondence authors: [sangil.kim@pusan.ac.kr](mailto:sangil.kim@pusan.ac.kr) ; ilhjung@pusan.ac.kr

**Proof of Theorem 1**: Let us consider the candidate functions as

|  | (A_1_) |
| --- | --- |

It is obvious that the function $V$is a positive-definite function. After taking the derivative of equation (A_1_) with respect to time and considering independent delay error, we obtain equation (A_2_):

|  | (A_2_) |
| --- | --- |

As the FHN system (equation (1)) has bounded trajectories, there exists a sufficiently small positive constant M>0, such that $\left|x_{i}\right|\leq M$ $(i=1,2,\cdots,n)$, Thus, we get

|  | (A_3_) |
| --- | --- |
|  | (A_4_) |

where

|  | (A_5_) |
| --- | --- |
|  | (A_6_) |

Here $\zeta_{ii}$, $\zeta_{(i+1)i}$, and $\zeta_{(i-1)i}$, where $i=1,2\cdots,n,$are given by

|  | (A_7_) |
| --- | --- |
|  | (A_9_) |

In this work, to prove that the matrix $\zeta$ is positive-definite, we consider a network of one thousand neurons that guarantee that the matrix $\zeta$ is positive-definite for the error system (equation (5)). According to the Lyapunov stability theory, the error system (equation (5)) is asymptotically stable about the origin, for any value of $0<g_{i}<0.1$. That is, a ring-structured network of *n*-identical and time-delayed coupled FHN neurons, with different gap junctions, under ionic gate disturbance and EES will achieve synchronization. Proof for the noisy unidirectional network of time-delayed FHN neuron is similar and therefore omitted.

**Proof of Theorem 2:** Let us consider the Lyapunov candidate function *V* as

|  | (A_10_) |
| --- | --- |

The Lyapunov function $V$ proposed in equation (A_10_) is a positive-definite function. Taking the time derivative of equation (A_10_) and considering independent delay error, we obtain equation (A_11_).

|  | (A_11_) |
| --- | --- |

As the time delayed FHN system (equation (6)) has bounded trajectories, a sufficiently small positive constant M>0 exists, such that $\left|x_{j}\right|\leq M$ $(j=1,2,\cdots,n)$. Thus, we get

|  | (A_12_) |
| --- | --- |

Equivalently,

|  | (A_13_) |
| --- | --- |

where

|  | (A_14_) |
| --- | --- |
|  | (A_15_) |

Here $\xi_{ii}$, $\xi_{(i+1)i}$, and $\xi_{(i-1)1}$, where $i=1,2\cdots,n.$ are given by

|  | (A_16_) |
| --- | --- |
|  | (A_17_) |

In this work, to prove that matrix $\xi$ is positive-definite, we consider a network of one thousand neurons that guarantee that the matrix $\xi$ is positive-definite for the error system (equation (9)). According to the Lyapunov stability theory, the error system (equation (9)) is asymptotically stable about the origin for any value of $0<g_{2j-1},g_{2j}<0.1$. That is, a ring-structured network of *n*-identical, time-delayed coupled FHN neurons, with different gap junctions, under ionic gate disturbance, and EES will achieve synchronization. Proof for the noisy bidirectional network of time-delayed FHN neurons is similar and therefore omitted.
